# Mobile Health Apps and Health Management Behaviors: Cost-Benefit Modeling Analysis

**DOI:** 10.2196/21251

**Published:** 2021-04-22

**Authors:** Rita Mano

**Affiliations:** 1 Department of Human Services University of Haifa Haifa Israel

**Keywords:** mobile health apps, health empowerment, health management behaviors, costs-benefits, mobile phone

## Abstract

**Background:**

Rising criticism about the risks associated with the use of mobile health apps necessitates a critical perspective to assess the use of these apps. A cost-benefit approach involving several moderating factors can be used to detect technology effects and individual-level push and pull factors related to health attitudes, lifestyle, and health management behaviors.

**Objective:**

We introduce a cost-benefit perspective to examine how health attitudes related to mobile health apps and health situational factors (health crises, health changes, and hospitalization) affect the likelihood of adopting lifestyle and health management behaviors among app users.

**Methods:**

The analysis is based on individuals’ reported use of mobile health apps. The sample included 1495 US adults aged over 18 years who were contacted by landline or cellphone. A total of 50.96% (762/1495) of the participants were women. A set of logistic regression models was used to predict lifestyle and health management behaviors among users considering variations in the extent of use, health attitudes, health situation, and socioeconomic characteristics.

**Results:**

The findings indicate that the proposed models were reasonably adequate. In all, 88.76% (1327/1495) of the cases were correctly classified regarding lifestyle behaviors, but only 71.97% (1076/1495) of the cases were correctly classified regarding health management behaviors. Although a large percentage of individuals changed their attitudes following the use of mobile health apps, only a small proportion adopted health management behaviors. The use of mobile health apps affected up to 67.95% (1016/1495) of the users for consultation and 71.97% (1076/1495) of the users for decision making. The model was effective for 88.76% (1327/1495) of the cases regarding lifestyle behaviors but only 71.97% (1076/1495) regarding health management behaviors. The moderating effect of regular use of mobile health apps significantly affects lifestyle (Wald=61.795; B=2.099; *P*<.005) but not health management behaviors (Wald=12.532; B=0.513; *P*=.01). These results collectively indicate that the use of mobile health apps for health management is partially effective.

**Conclusions:**

The use of mobile health apps is a main route to instigate the process of health empowerment and shape health attitudes. However, an accurate assessment of the effectiveness of mobile health apps necessitates distinguishing between lifestyle and health management behaviors and adopting a cost-benefit approach because individuals facing health concerns, such as a chronic disease, health emergency, health crisis, or health change, consider their affordances and situational effects. These moderators generate a push and pull framework in the decision-making process that balances the costs and benefits of use.

## Introduction

### Background

Finding new ways to support and care for various groups of people living at home has become a challenge for health care providers [1], brought about by the growth of the aging population as well as the shortage of hospital beds [1,2]. This challenge has been partly addressed by the introduction of technology-based tools and services [2] and has led many countries to apply information technology to telemedicine care services [3]. Among these tools, mobile health apps provide general support in the areas of medical education [4], preventative health care [5], health monitoring [6], and illness management [7]. More than 100,000 mobile health apps are available on smartphones [4]. Approximately 3 to 4 billion smartphone and tablet users use mobile health apps to download and update health fitness programs, contact health care professionals, and monitor health conditions, and most users access at least one health-related app [8].

Indeed, mobile health apps play a major role in self-management and care at home. Few existing studies have explored the variations in using mobile phones for health-related issues while on the go, and some studies have begun to report user feedback on specific apps [1], mostly showing that these technological advances [2] have enabled better health care services to be provided to the public [9]. Not surprisingly, mobile health apps attract the attention of institutional health care providers [8,10] for various purposes, such as improving treatment, diagnosing early symptoms, providing faster responses, accessing medical data and decision support systems, increasing digital health literacy, and accentuating support on social platforms [11].

Many studies have assessed the feasibility, functionality, clinical utility, benefits, and risks of mobile health apps [12-16]. Evidence indicates that mobile health apps are effective in providing feedback and improving goal setting and self-monitoring in eating disorders [17], alcohol use disorders [18], and attempts to stop smoking [19]. They are also used to encourage physical activity [20] and provide psychotherapy [15]. The demand for home care services has grown over the last decades [9,21] to support individuals and diverse groups [2], including the aging and chronically ill people [11], to better manage their health at home [4]. However, some of these studies have also indicated that the focus on specific groups led to a missed opportunity to address how users facing health-related emergencies put off further use of mobile health apps [21-25].

First, technology skills vary [26], as do the purposes and extent of technology use [27,28]. Second, health management behaviors involve different levels of uncertainty and vulnerability [28] or perceived threats [29,30]. Third, health attitudes do not necessarily coincide with health management behaviors [31], as issues of functionality may not necessarily lead to lifestyle and health management behaviors [32]. Finally, sociodemographic variations are important when considering both the use of mobile health apps and health management behaviors [33,34]. This is why we need to distinguish between lifestyle health management behaviors, such as increasing daily vitamin intake and engaging in a physical fitness program, and more complex health care management behaviors, such as those related to the management of serious health concerns [12,35] considering the different needs and affordances of individuals.

In this study, we address these concerns. We consider the possibility that even though mobile apps are highly accessible and exert a general beneficial effect on health attitudes and empowerment, their potential to encourage health management behaviors is limited due to the limited consideration of individual health situations and affordances. We examine how variations in the use of mobile health apps enhance or restrain the adoption of lifestyle and health management behaviors among individuals experiencing health concerns [9,11,34] and health crises.

The shift from *mechanical to informational* medicine [36] has placed a growing responsibility on individuals regarding health concerns [37] and urged them to increase their own health awareness through access to web-based health information [38] and health services [39]. Mobile health apps increase health awareness and instigate health management behaviors by causing individuals to adhere to new health routines and improve existing ones [40]. Three major theoretical directions enable an integrative approach: (1) technology-human interaction models, (2) health empowerment (HE) and health belief model (HBM), and (3) the social diversification hypothesis (SDH) [33].

### Technology-Human Interaction Models

The technology acceptance model (TAM) [41] focuses on factors associated with the use of internet communication technology (ICT). TAM assumes that variations in the acceptance of computerized technology reflect a set of facilitating conditions, including expected effort, performance, and social influence [42,43]. TAM suggests that individuals will adopt technology when its perceived usefulness and perceived ease of use are high, and ICT use is likely to shape a new set of attitudes regarding technology’s potential to contribute to health purposes [44,45]. The *perceived functionality* of mobile health apps will increase the level of use of the mobile health apps and the need to update such apps [46]. Health adoption models test these assumptions.

### The HE and HBM Perspective

The HE perspective introduced the notion of health efficacy and the right to express health aspirations, thus enabling individuals to develop critical awareness about their existing health conditions [47-49]. The HE model complements assumptions from communications and computer-mediated models and provides specific hypotheses about the effect of individual health-related conditions on health changes. Individuals who learn and internalize aspects of health and disease and develop health-related consciousness are more likely to express health-related aspirations and expectations, and these individuals develop the confidence to adhere to a more focused approach to health concerns, making them more willing to use mobile health apps [50]. Moreover, a rational consumer choice approach will motivate individuals to seek even more information and compare multiple sources of information before making health decisions [49].

HBM applies the concepts of self-efficacy and HE. Initially, HBM suggested that beliefs and attitudes moderate the impact of technology on health management behaviors among individuals concerned with health issues [51]. Later, HBM focused on the perceived benefits or barriers stemming from taking action to prevent diseases or disorders [28]. The relative weight of benefits versus barriers affects the likelihood of taking preventive action. When barriers are perceived to be high, individuals are less likely to engage in healthy lifestyle behaviors [52]. HBM was applied to predict helmet use [53], improve driving [27], improve adherence to treatment [28], and improve communication about health concerns [44]. Both HE and HBM suggest that individuals will be more willing to play an active role in preventing, treating, and following up on health issues for themselves and others [49,54]. Hence, we hypothesize the following:

- *H1*: Greater use of mobile health apps will increase the likelihood of a change in approach when addressing a health concern.
- *H2*: Greater use of mobile health apps will increase the likelihood of making a decision to address a health concern.
- *H3*: Greater use of mobile health apps will increase the likelihood of asking a health provider new questions or seeking a second opinion from another doctor.

Nonetheless, neither the HE nor the HBM model provides the necessary assumptions to tap into factors associated with choices and behaviors when individuals face a set of health-related situations.

### The SDH Perspective

SDH addresses the possible outcomes of inequalities in the use of ICT devices on additional aspects of life, such as health [55]. ICT devices serve as a major vehicle for overcoming environmental barriers, both geographic and temporal. Nevertheless, their use is often affected by the (1) costs involved in the acquisition and use of ICT and mobile devices [56]; (2) technology skills necessary to use such devices [57]; (3) individuals’ beliefs, attitudes, goals, and plans; and (4) differences in their socioeconomic background [58]. These socioeconomic characteristics, including age, gender, ethnic background, education, and income, are proxies for the potential to (1) use and (2) apply technology devices [33,55]. Similarly, recent studies indicate that aging individuals are less likely and women are more likely to use and capitalize on technology to adopt lifestyle and health management behaviors [34]. Hence, we hypothesize the following:

- *H4*: Greater use of mobile health apps will increase the likelihood of adopting lifestyle health management behaviors after controlling for variations in socioeconomic factors and health attitudes.
- *H5*: Greater use of mobile health apps will increase the likelihood of adopting health management behaviors after controlling for variations in socioeconomic factors and health attitudes.

Mobile health apps may inspire individuals to reshape their health attitudes. Nonetheless, individuals may also *critically* evaluate the functionality of mobile health apps and dismiss the use of mobile health app guidelines and programs [49,54]. A perceived threat that might otherwise motivate individuals to adopt lifestyle health management behaviors [30,44] may cause individuals to restrain from the use and influence of mobile health apps [59].

### Health Behaviors: The Concept of Affordances

Overall, the HE and HBM models [60], and to some extent SDH [33], assume that rational health management behaviors emerge when individuals develop empowering attitudes regarding a health concern. However, these assumptions are based on shaky ground. First, individuals may not necessarily behave rationally, especially when many additional factors come into play. Second, individuals are more likely to capitalize on virtual health information regarding lifestyle but not on health management [59]. To clarify these points, we addressed the role of affordances [61] in health management behaviors.

The concept of *affordance* captures the beneficial or injurious aspect of objects and is relative in terms of how well objects fit an individual situation. The strength of affordances lies in the individual’s perceptions regarding the need to weigh one’s action *possibilities* [62]. The term a*ffordances* denotes the need to address everyday objects together with their features and functions. Individuals using a device are seldom preoccupied with its objective qualities because these objective features and functions do not necessarily fit users’ needs. A lack of fit shapes individuals’ perceived affordances and generates the need to assess the costs and benefits of using apps. As a result, individuals use a push and pull framework in their decision-making process before acting on the content of the ICT medium [34]. A set of personal situations may encourage or discourage individuals from developing favorable health attitudes and adopting health management behaviors. Hence, we hypothesize the following:

- *H6*: Greater use of mobile health apps will increase the likelihood of lifestyle behaviors after controlling for variations in mobile health app use and health attitudes.
- *H7*: Greater use of mobile health apps will increase the likelihood of health management behaviors after controlling for variations in mobile health app use and health attitudes.

Technology devices such as mobile health apps are reported to fall short of their intended purposes [63-65] because, in practical terms, individuals assess their situation and apply a push and pull decision-making process [66].

### The Push and Pull Perspective: A Situational Approach to Health Behavior

The push-pull perspective analyzes the migration decisions [66]. It highlights the need to identify the best *destination* option during migration while considering a set of factors that may *threaten* the outcome of the migration. Favorable conditions *push* individuals in a specific direction toward a specific location, whereas less favorable conditions *pull* them away. By applying the push-pull perspective in health, we can assume that individuals’ health management behaviors depend on the way they relate to their specific health situation, especially when it involves a perceived threat or risk [15,44]. In the process, users will consider adopting mobile health apps according to their specific situation regarding a health concern, especially when it manifests in a medical emergency or an unexpected health change. This *situational* health context will ultimately shape their perceived affordances regarding the use of mobile health apps and affect their health management behaviors [62,67,68]. Individuals may then consider their affordances in terms of the potential of mobile health apps to support their needs in light of their situation. When these affordances are costly, individuals may not be willing to use mobile health apps, especially individuals diagnosed with a chronic condition [39]. Hence, we hypothesize the following:

- *H8*: Use of mobile health apps will increase the likelihood of lifestyle behaviors after controlling for situational effects.
- *H9*: Use of mobile health apps will increase the likelihood of health management behaviors after controlling for situational effects.

### Objectives

This study aimed to investigate the variations in health attitudes and behaviors of individuals using mobile health apps. We conducted an analysis of smartphone users to explore the extent to which the use of mobile health apps enhances or restrains the adoption of health management behaviors among individuals experiencing different situational health concerns. We address their existing experiences of using health-related smartphone apps and their health management behaviors following the currently available or future apps. We sought to determine the extent of use and behaviors relevant to lifestyle and health management. We also considered that a set of moderating push and pull factors, including the diagnosis of medical health and the occurrence of a health emergency crisis, may lead to disinclination to use the apps.

## Methods

### Sample

This study draws on a secondary analysis of the data released by Princeton [69]. The sample was taken from a national tracking survey of 8323 individuals aged over 18 years and contacted by landline or cellphone. The analysis is based on individuals’ reported use of mobile health apps (N=1495). The sample comprised 50.96% (762/1495) women; 60.6% (921/1354) were married or cohabitating, 41.33% (618/1495) were parents of children living at home, 29.69% (444/1495) had less than a college degree, and 24.15% (361/1495) earned less than US $30,000. A total of 79.66% (1191/1495) of the sample reported using a single health app, and 20.06% (300/1495) of the sample reported using more than one app (Multimedia Appendix 1).

### Dependent Variables

#### Health Behaviors

Health behaviors manifest in two different ways: (1) lifestyle behavior: do you currently keep track of your own weight, diet, or exercise routine? (1=yes) and (2) health management behavior: do you happen to track your own blood pressure, blood sugar, sleep patterns, headaches, or any other indicator? (1=yes).

#### Health Attitudes

The use of mobile health apps influenced the following: (1) approach: has tracking this health indicator changed your overall approach to maintaining your health or the health of someone you help take care of? (1=yes), (2) decision making: has tracking this health indicator affected a decision about how to treat an illness or condition? (1=yes), and (3) consulting: has the use of mobile health apps led you to ask a doctor new questions or to seek a second opinion from another doctor (1=yes).

#### Independent Variables

The independent variables refer to the use of mobile health apps: (1) number of apps used: what kind of health apps do you currently have on your phone? Respondents replied to the question 10 times for 10 uses. We used the first 4 counts reporting 4 different types of health concerns. The range is from only one use to four uses, (2) updates (1=yes), and (3) update frequency (1=every day).

### Control Variables

#### Socioeconomic Characteristics

An important role to the use of apps for health purposes is the role assigned to socioeconomic variations. There are 5 key variables, which have been described below.

##### Age

Age is a proxy for technology skills and the likelihood of chronic illness (18-85 years). Studies have shown that older individuals perform more poorly than young people in using internet browsers, finding search engines, and navigating the internet [57]. Older people often experience more difficulties using technology than younger people [33], which may affect both use and outcomes among older age groups [70,71]. Moreover, health usually deteriorates with age [72], so age may be an important motivation for seeking health-related information and engaging in health-related discussions [73].

##### Gender

Consistent findings indicate that women use the internet for health purposes more than men do [33,34], reflecting their social function of family caregivers [33] and health managers [74]. Men were also found to have lower odds of using health sites and web-based consultations [75] (1=male).

##### Marital Status

Married or cohabitating individuals are reported to be more likely to use web-based health services [59] and consult web-based rankings or reviews [75] (1=yes).

##### Education

Education increases the likelihood of health literacy and the ability to understand medical information, including drug prescriptions, the etiology of diseases, and risks. Better cognitive skills, attributed to highly educated individuals, lead to a better evaluation of health information [59]. Therefore, more educated individuals may want to use technology for health-related concerns more than less educated individuals (ranging from 1=no formal education to 10=PhD).

##### Income

How much did you earn last year? Studies on inequalities in the use of web-based health information have found differences between groups based on their socioeconomic status. The likelihood of searching for web-based health information was inversely associated with income (ranging from 1=less than US $10,000 to 6=less than US $150,000).

#### Situational Effects

Individuals’ health management includes several specific conditions that may affect the use of apps for health purposes: (1) chronic disease: previous studies have shown that those who report having a chronic illness are more likely to seek medical information and participate in online health-related forums [40,67]; (2) health crisis: in the last 12 months, have you personally faced a serious medical emergency or crisis (1=yes); (3) health emergency: in the last 12 months, have you personally gone to the emergency room or have been hospitalized unexpectedly (1=yes); and (4) health change: in the last 12 months, have you personally experienced any significant change in your physical health, such as gaining or losing a lot of weight, becoming pregnant, or quitting smoking (1=yes).

### Strategy Analysis

To examine the effect of technology use on (1) health attitudes and (2) health management behaviors, we implemented the following steps.

First, we provide a general description of the distribution of the sample across the study variables (Multimedia Appendix 1).

Second, we tap into an overall estimation of the impact of the model’s independent and control variables on the dependent variable (health attitudes following the use of mobile health apps) using the classification tables of a logistic regression procedure. We estimate the correctly classified cases, which cover both successful and failed cases. We present the results separately for the effects of mobile health app use on health attitudes (Table 1) and on lifestyle and health management behaviors (Table 2).

**Table 1:** Logistic regression summary models predicting the number of correctly classified cases for the model that predicts the influence of mobile health apps on health attitudes.

Observed effects		Participants		
		Number of correctly predicted cases		Percentage of correctly predicted cases
		False	True	
**Influenced health approach**				
	False	377	294	56.2
	True	182	638	77.8
	Overall percentage	N/A^a^	N/A	68.1
**Influenced health decision**				
	False	844	98	89.6
	True	321	228	41.6
	Overall fit	N/A	N/A	71.9
**Influenced consulting**				
	False	761	145	84.0
	True	334	252	43.0
	Overall fit	N/A	N/A	67.9

^a^N/A: not applicable.

**Table 2:** Logistic regression summary models and percentage of correctly classified cases predicting likelihood of lifestyle and health management behaviors following the use of mobile health apps.

Observed effects		Participants				
		Number of correctly predicted cases				Percentage of correctly predicted cases
		False		True		
**Lifestyle behavior**						
	False	132		107	55.2	
	True	60		1192	95.2	
	Overall fit	N/A^a^		N/A	88.8	
**Health management behavior**						
	False		807	145	84.8	
	True		273	267	49.4	
	Overall fit		N/A	N/A	72.0	

^a^N/A: not applicable.

Third, we explored the direct impact of mobile health apps’ use on lifestyle and health management behaviors by using logistic regression. To this end, we proceeded systematically. First, we introduced the set of variations in mobile health apps’ use (number of mobile health apps and update frequency). Second, we added the impact of variations in health attitudes following mobile health apps’ use. Subsequently, we inserted socioeconomic effects and situational effects. This hierarchical systematic method enables us to assess the extent to which variables in each set of predictors increase or decrease the likelihood of predicting lifestyles (Table 3) and health management behaviors (Table 4).

**Table 3:** Logistic regression coefficients predicting health attitudes following the use of mobile health apps.

Variables affecting health attitudes				B		SE		Wald		Significance (*P* value)		Explained (B)	
**Approach regarding a health concern**													
	**Mobile health apps’ use**												
		Number of health apps = −1	−0.448^a^		0.112		16.046		<.001		0.639		
		Number of health apps = +1	0.247		0.174		2.006		.16		1.280		
		Updates frequency	1.40^a^		0.128		120.447		<.001		4.054		
	**Socioeconomic factors**												
		Sex: 1=male	0.000		0.006		0.004		.95		1.000		
		Married or cohabitation	0.772^a^		0.126		37.351		<.001		2.163		
		Parenthood	0.034		0.035		0.961		.33		1.035		
		Education	0.048		0.152		0.099		.75		1.049		
		Income	−0.155^a^		0.037		17.252		<.001		0.857		
**Decision regarding a health concern**													
	**Mobile health apps’ use**												
		Number of health apps = −1	−0.801^a^		0.138		33.804		<.001		0.449		
		Number of health apps = +1	0.794^a^		0.170		21.787		<.001		2.212		
		Updates frequency	−0.164		0.127		1.664		.197		0.849		
	**Socioeconomic factors**												
		Sex: 1=male	0.009		0.006		2.420		.12		1.010		
		Married or cohabitation	0.417^a^		0.128		10.562		.001		1.518		
		Parenthood	0.054		0.036		2.253		.13		1.055		
		Education	0.232		0.157		2.184		.14		1.261		
		Income	−0.120^a^		0.039		9.749		.002		0.887		
**Consulting regarding a health concern**													
	**Mobile health apps’ use**												
		Number of health apps = −1	0.197		0.112		3.071		.08				1.217
		Number of health apps = +1	1.749^a^		0.174		100.893		<.001				5.748
		Updates frequency	−0.348^a^		0.125		7.762		.005				0.706
	**Socioeconomic factors**												
		Sex: 1=male	−0.008		0.006		1.580		.21				0.992
		Married or cohabitation	−0.195		0.126		2.381		.12				0.823
		Parenthood	0.056		0.035		2.589		.11				1.058
		Education	−0.048		0.156		0.094		.76				0.953
		Income	−0.142^a^		0.038		14.036		<.001				0.867

^a^*P*<.001.

**Table 4:** Logistic regression coefficients predicting lifestyle and health management behaviors following the use of mobile health apps.

Characteristics		Lifestyle behavior						Health management behavior				
		B	SE	Wald	Significance (*P* values)	Explained (B)	B		SE	Wald	Significance (*P* values)	Explained (B)
**Mobile health app use**												
	Number of mobile apps = −1	−0.863^a^	0.187	21.295	<.001	0.422	0.220		0.124	3.154	.08	1.246
	Number of mobile apps = +1	−1.827^a^	0.330	30.725	<.001	0.161	0.042		0.200	0.044	.83	1.043
	Frequency of updates	2.099^a^	0.267	61.795	<.001	8.162	0.513		0.145	12.532	<.001	1.670
**Health attitudes**												
	Approach	1.493^a^	0.263	32.110	<.001	4.450	0.230		0.155	2.189	.14	1.258
	Decision	0.865^a^	0.283	9.333	.002	2.374	0.914		0.155	34.915	<.001	2.494
	Consulting	2.713^a^	0.322	70.820	<.001	15.07	0.481		0.154	9.796	.002	1.618
**Socioeconomic effects**												
	Age	−0.070^a^	0.011	44.445	<.001	0.932	0.042		0.007	35.855	<.001	1.043
	Sex: 1=male	−01.736^a^	0.244	50.567	<.001	0.176	−0.406		0.143	8.094	.004	0.666
	Married: 1=yes	−0.043	0.060	0.516	.47	0.958	0.238		0.039	36.962	<.001	1.269
	Parent: 1=yes	−0.144	0.250	0.332	.56	0.866	0.595		0.171	12.175	<.001	1.813
	Education	0.641^a^	0.069	85.811	<.001	1.898	−0.094		0.042	4.944	.03	0.911
	Income	0.002	0.003	0.290	.59	1.002	0.002		0.002	0.618	.43	1.002
**Situational effects**												
	Chronic disease	−1.35^a^	0.239	32.221	<.001	0.257	1.007		0.142	50.471	<.001	2.737
	Emergency	−1.842^a^	0.602	9.367	.002	6.312	−1.012		0.280	13.101	<.001	0.363
	Health crisis	−0.751^a^	0.343	4.795	.03	0.472	1.086		0.208	27.256	<.001	2.962
	Health change	−0.699^a^	0.254	7.583	.006	0.497	−0.679		0.168	16.290	<.001	0.507

^a^*P*<.001.

## Results

### Testing the Overall Fit of a Push and Pull Model in Predicting Health Attitudes and Health Behaviors

First, we tested how well the proposed model enabled us to correctly classify the examined cases. The findings indicate that the proposed models are reasonably adequate and make it possible to classify the examined cases correctly for health attitudes (up to 1076/1495, 71.97%) following the use of apps. The overall percentage of correctly predicted cases indicates that the use of mobile health apps affects up to 67.95% (1016/1495) individuals for consultation and 71.97% (1076/1495) for decision making. The model was effective for 88.76% (1327/1495) of the cases regarding lifestyle behaviors but only for 71.97% (1076/1495) of the cases regarding health management behaviors.

A closer look at the positive outcomes shows that a higher level of involvement in the reaction, which ranged from a mere attitude to a practical behavior, decreased the effectiveness of mobile health apps. Although a large percentage of individuals (1163/1495, 77.79%) changed their attitudes following the use of mobile health apps, only a small proportion (738/1495, 49.36%) used them for health management behaviors and even less (642/1495, 42.94%) sought out a second opinion. Therefore, the results indicate that using mobile health apps is generally less effective in generating higher HE than expected, especially after considering situational effects.

### Mobile Health Apps and Health Attitudes

#### Extent of Use

The findings in Table 3 suggest that an increase in mobile health apps’ use does not have a uniform effect on health-related attitudes. In addition, the number of apps used is likely to have both positive and negative effects. For example, although the use of a limited number of mobile health apps can decrease the likelihood of changing the user’s approach (Wald=16.046; B=−0.448), only the use of more than one app increases the likelihood of taking steps to seek further consultation (Wald=100.893; B=1.749) as well as to make a decision (Wald=21.787; B=0.794).

#### Updates

Similarly, an increase in the frequency of updates can increase the likelihood of changing a user’s approach (Wald=120.447; B=1.4), but it can also decrease the likelihood of seeking further consultations from a health provider (Wald=7.762; B=−0.348). Individuals with specific health concerns are more likely to crosscheck information or look for multiple health concerns. These results clearly point to the possibility of distress following the excessive use of mobile health apps in terms of information overload, similar to the technology fatigue syndrome already apparent in the use of email-based communication and the differential effects of digital communication on individuals’ well-being [76]. To explore the source of these differences and in line with SDH [33], we next examined socioeconomic effects.

#### Socioeconomic Effects

The most impressive findings among the socioeconomic effects are the negative effects of income level and marital status. The higher the income, the less likely it is that users will be affected by mobile health apps in terms of approach (Wald=17.252; B=−0.155), decision making (Wald=9.749; B=−0.120), or consulting (Wald=14.036; B=−0.142). The significant effect of higher income as a pull factor on the effect of mobile health apps indicates that income may increase the likelihood of using less technology for both leisure and health concerns. Being in a spousal relationship increases the likelihood of a changed approach (Wald=37.351; B=0.772) to decision making (Wald=10.562; B=0.417), but it has no significant effect on consulting regarding a health concern (Wald=2.381; B=−0.195). The results indicate that individuals in spousal relationships are more likely to address the health concerns of their spouse as well as their own.

### Mobile Health Apps’ Use and Situational Effects

To explore the direct impact of technology on (1) lifestyle and (2) health management behaviors, we proceed in a stepwise manner. The stepwise method enables us to explore the extent to which variables in each set of predictors increase or decrease the likelihood of predicting health lifestyle and health management behaviors. First, we introduced variations in mobile health apps’ use—the number of mobile health apps and update frequency. Second, we predicted variations in health attitudes following the use of mobile health apps. Third, we introduced socioeconomic variables, controlling for both mobile health apps’ use and health attitudes.

In the final step, we introduced *situational* variables to assess the extent to which the use of mobile health apps is beneficial to lifestyle and health management behaviors.

#### Use of Mobile Health Apps

The findings in Table 4 indicate that the use of mobile health apps (eg, the number of mobile health apps and updating frequency) has a differential effect on health management behaviors. More specifically, using a greater number of mobile health apps significantly decreases the likelihood of lifestyle health management behaviors among users (Wald=21.295; B=−0.863), but it has no significant effect on health management behaviors (Wald=3.154; B=−0.220). However, regular updates increase both lifestyle (Wald=61.795; B=2.099) and health management (Wald=12.532; B=0.513) behaviors.

#### Health Attitudes

Next, we examined the effects of health attitudes on health management behaviors. An empowering change of approach (Wald=32.110; B=1.493), making a decision (Wald=9.333; B=0.865), and seeking further consultation (Wald=70.820; B=2.713) regarding a health concern following the use of mobile health apps increase the likelihood of lifestyle health management behaviors. Similar effects are evident regarding health management behaviors, with the exception of change in approach. Making a decision (Wald=34.915; B=0.914) and seeking further consultation (Wald=9.796; B=0.481) regarding a health concern following the use of mobile health apps increase the likelihood of health management behaviors.

#### Socioeconomic Effects

The most prominent findings indicate the mixed effects of socioeconomic variables in predicting lifestyle and health management behaviors. Older adults (Wald=44.445; B=−0.070) and men (Wald=50.567; B=−1.736) were less likely to instigate lifestyle health management behaviors following the use of mobile health apps. Furthermore, educated users were more likely to pursue lifestyle health management behaviors following the use of mobile health apps (Wald=85.811; B=0.641). The combined effect of the use of mobile health apps and socioeconomic factors clearly indicates that mobile health apps have an empowering effect on both lifestyle and health management behaviors among users. Nonetheless, the extent to which these sets of factors remain effective necessitates considering situational effects that can possibly reverse this general trend.

#### Situational Effects

Overall, the results pointing to the influence of situational effects on lifestyle and health management behaviors are indicative of the significance of such effects on health management behaviors. Situational effects have mixed effects. They can have a negative effect on lifestyle health management behaviors and less on health management behaviors. A chronic disease (Wald=32.221; B=−1.359), a health emergency (Wald=9.367; B=−1.842), a health crisis (Wald=4.795; B=−0.751), and a health change (Wald=7.583; B=−0.699) all decrease the likelihood of adopting lifestyle health management behaviors. Moreover, the effect of situational factors on health management behaviors is not uniform. Chronic disease (Wald=50.472; B=1.007) and health crises (Wald=27.256; B=1.086) increase the likelihood of health management behaviors. In contrast, a health emergency (Wald=13.101; B=−1.012) and a health change (Wald=16.290; B=−0.679) decrease the likelihood of health management behavior.

These results provide the following conclusions. First, it is evident that situational effects create some kind of general perception of risk [15] because they inhibit the effective impact of mobile health apps on lifestyle behaviors, such as weight loss or physical activity. Second, there is apparently a difference in the way individuals perceive the *threat* related to their situation. Chronic diseases, but not health crises, often manifest in the form of health management *routine* [77]. In this case, the use of mobile health apps helps to address the health concerns of individuals who are already aware of their health condition. However, in the case of an emergency or a sudden change in health, mobile health apps may become irrelevant and possibly risky [8].

## Discussion

### Principal Findings

In this study, we assessed the impact of mobile health apps on health attitudes, lifestyle health management behaviors, and health management behaviors. We adopted a cost-benefit approach and applied the push-pull perspective to introduce a set of *situational* factors including health crises, changes in health condition, and sudden hospitalization. We considered the possibility that situational health factors affecting individual affordances may, in some cases, enhance (push) the adoption of lifestyle and health management behaviors following the use of mobile health apps, whereas in others, they may restrain (pull) this adoption. Overall, the classification model indicates that mobile health apps are only partially effective because a set of *situational* effects moderates the link between the use of mobile health apps and health management behaviors. In fact, although a large percentage of individuals change their health-related attitudes following the use of mobile apps, a much smaller portion adopts health management behaviors. These findings support most of the proposed hypotheses.

First, technology use clearly affects health attitudes, increasing the likelihood that mobile health apps will change attitudes and causing users to seek out advice about health concerns based on the knowledge acquired through mobile health apps, but it is also possible that the users may go a little *overboard* and become confused and distressed [76]. Second, although positive attitudes increase the likelihood of developing empowering health attitudes [53], these attitudes may not necessarily prompt users to actually engage in health management behaviors. Indeed, the occurrence of situational effects, such as a sudden change in health, health crises, and hospitalization generate *different realities* that shape individuals’ affordances and define the limits of their own cost-benefit framework that accounts for the push and pull factors and encourages or discourages health management behaviors [8]. As a result, for individuals who experience health-related concerns, tailored programs are less appealing because they have specific needs or even face health risks.

These findings help in assessing similar conclusions in recent studies [6,8,69] and necessitate considering *situational* effects in an individual’s health management behavior in both lifestyle and health management behaviors. Therefore, the prediction of health management behaviors following the use of mobile health apps aiming to increase the likelihood of adopting effective health management behaviors should be assessed within a push and pull framework.

### Strengths and Limitations

The use of mobile apps for health purposes represents an important breakthrough in ICT. The availability of mobile health apps affects individuals wishing to enhance their levels of HE and improve their health routine. Individuals use these apps for various health purposes. These include lifestyle behaviors, such as quitting smoking, adhering to physical fitness programs, and accessing health services, and health management behaviors, such as adhering to sugar and blood pressure monitoring, cancer and heart disease management, and psychotherapy support. However, existing studies supporting the beneficial effects of mobile health apps have focused mostly on specific health groups and less on a wide range of individuals with or without health concerns. As a result, there is little evidence of a cross-sectional comparison of the usefulness of mobile health apps. This is especially important considering that health institutions and professionals report that they rely increasingly on the use of mobile health apps to increase health awareness and promote adherence to health management practices.

### Conclusions

We conclude that the effect of mobile health apps on health management behaviors should intersect with both the objective qualities of those apps and health situational factors and not just induce empowering health attitudes [61]. Designers of mobile health apps should take into account the effect of possible barriers to effective use of apps. Acknowledging these barriers will assist to develop in-depth insights into how and why health lifestyle and health management behaviors develop following the use of mobile health apps. These insights will in turn assist individuals who depend on the effective use of these apps to address frail health conditions and attain effective home care support.

## Appendix

Multimedia Appendix 1 Distribution of central variables (N=1491).

## Abbreviations

HBM: health belief model
HE: health empowerment
ICT: internet communication technology
SDH: social diversification hypothesis
TAM: technology acceptance model
