# Supplementary material for: Mobile Health Apps and Health Management Behaviors: Cost-Benefit Modeling Analysis
Source: JMIR Hum Factors. 2021 Apr 22;8(2):e21251. doi: 10.2196/21251 (PMC8103300; doi:10.2196/21251)
Supplement: Multimedia Appendix 1 [file humanfactors_v8i2e21251_app1.pdf]

Appendix 1:
Distributions of central variables (N=1491)

HEALTH BEHAVIORS	N	Valid %
Lifestyle behavior (1=yes)	540	36.2
Health management behavior (1=yes)	1252	63.8
MOBILE HEALTH APPLICATION USE		
Number of mobile health apps used - 1	1153	77.3
Number of mobile health apps used + 1	287	23.7
Mobile health apps update (1=yes)	681	45.7
High Frequency for mobile health apps update (1=yes)	227	15.2
HEALTH ATTITUDES		
Mobile health apps changed approach for health concern (1=yes)	820	55.0
Mobile health apps affected decision for health concern (1=yes)	549	36.8
Mobile health apps led to further consulting (1=yes)	585	39.3
SITUATIONAL EFFECTS		
Chronic disease (1=yes)	487	32.6
Health emergency experience (1=yes)	129	8.6
Health crisis experience (1=yes)	256	17.1
Health change (1=yes)	428	28.7
SOCIOECONOMIC		
Sex: 1=male	613	41.1
Marital status: 1=Married/ living with a partner	792	53.1
Parenthood : children less than 18 at home	606	40.7
Education less than college	449	43.2
Income less than $40,000	184	29.6
